# Intervention Services for Autistic Adults: An ASDEU Study of Autistic Adults, Carers, and Professionals’ Experiences

**DOI:** 10.1007/s10803-021-05038-0

**Published:** 2021-05-08

**Authors:** Martina Micai, Antonio Ciaramella, Tommaso Salvitti, Francesca Fulceri, Laura Maria Fatta, Luise Poustka, Robert Diehm, Georgi Iskrov, Rumen Stefanov, Quentin Guillon, Bernadette Rogé, Anthony Staines, Mary Rose Sweeney, Andrew Martin Boilson, Thora Leósdóttir, Evald Saemundsen, Irma Moilanen, Hanna Ebeling, Anneli Yliherva, Mika Gissler, Tarja Parviainen, Pekka Tani, Rafal Kawa, Astrid Vicente, Célia Rasga, Magdalena Budişteanu, Ian Dale, Carol Povey, Noelia Flores, Cristina Jenaro, Maria Luisa Monroy, Patricia García Primo, Tony Charman, Susanne Cramer, Christine Kloster Warberg, Ricardo Canal-Bedia, Manuel Posada, Maria Luisa Scattoni, Diana Schendel

**Affiliations:** 1 Research Coordination and Support Service, Istituto Superiore Di Sanità, Viale Regina Elena 299, 00161 Rome, Italy; 2 Department of Child and Adolescent Psychiatry and Psychotherapy, University Medical Center Göttingen, Gottingen, Germany; 3 Department of Child and Adolescent Psychiatry and Psychotherapy, Medical University of Vienna, Wien, Austria; 4 Institute for Rare Diseases, Plovdiv, Bulgaria; 5 Faculty of Public Health, Department of Social Medicine and Public Health, Medical University of Plovdiv, Plovdiv, Bulgaria; 6 Université Toulouse Jean Jaurès, CERPPS, Toulouse, Occitanie France; 7 School of Nursing, Dublin City University, Psychotherapy & Community Health, Dublin, Ireland; 8 The State Diagnostic and Counselling Centre, 200 Kópavogur, Iceland; 9 Clinic of Child Psychiatry, University and University Hospital of Oulu, Oulu, Finland; 10 Medical Faculty, Oulu University Hospital, Oulu, Finland; 11 Logopedic Child Language Research Center, University of Oulu, Oulu, Finland; 12 Finnish Institute for Health and Welfare, Helsinki, Uusimaa Finland; 13 Research Centre for Child Psychiatry, University of Turku, Turku, Finland; 14 Division of Family Medicine, Department of Neurobiology, Care Sciences and Society, Karolinska Institute, Stockholm, Sweden; 15 Finnish Association for Autism and Asperger’s Syndrome, Helsinki, Uusimaa Finland; 16 Department of Psychiatry, University of Helsinki, Helsinki, Finland; 17 Faculty of Psychology, University of Warsaw, Warsaw, Poland; 18 Center for Biodiversity, Functional and Integrative Genomics, Instituto Nacional de Saúde Doutor Ricardo Jorge, Lisbon, Portugal; 19 Development in Pathology and Biomedical Sciences, “Victor Babeş” National Institute for Research, Timisoara, Romania; 20 The Centre for Autism, National Autistic Society, London, UK; 21 Dpto. Personalidad, Evaluación Y Tratamiento Psicológicos, INICO - Instituto Universitario de Integración en La Comunidad University of Salamanca, Salamanca, Spain; 22 Departamento de Psicología Evolutiva Y de La Educación, INICO - Instituto Universitario de Integración en La Comunidad University of Salamanca, Salamanca, Spain; 23 Institute of Rare Diseases Research, Instituto de Salud Carlos III, Madrid, Spain; 24 Institute of Psychiatry, Kings College London, London, UK; 25 Department of Public Health, Aarhus University, Aarhus, Denmark; 26 Lundbeck Foundation Initiative for Integrative Psychiatric Research, iPSYCH, Aarhus, Denmark; 27 Department of Economics and Business, National Centre for Register-Based Research, Aarhus University, Aarhus, Denmark

**Keywords:** Autism Spectrum Disorder, Adults, Interventions, Services

## Abstract

The Autism Spectrum Disorders in the European Union (ASDEU) survey investigated local services’ use experiences of autistic adults, carers and professionals with interventions for autistic adults. The majority of the 697 participants experienced recommended considerations prior to deciding on intervention and during the intervention plan and implementation. Psychosocial interventions were the most commonly experienced interventions, while pharmacological interventions NOT recommended for core autistic symptoms were reported by fairly large proportions of participants. Family interventions were experienced slightly more commonly by carers than adults or professionals. Less than the 26% of autistic adult responders who had experienced challenging behaviors reported receiving an intervention to change them. These results provide insights for improving gaps in service provision of interventions among autistic adults.

**Supplementary Information:**

The online version contains supplementary material available at 10.1007/s10803-021-05038-0.

## Introduction

The autistic condition is characterized by deficits in social communication and interaction, and restricted/repetitive repertoires of behaviors, interests and activities (Autism Spectrum Disorder, American Psychiatric Association, 2013) and usually persists into adulthood (Howlin et al., 2004, 2013; Woolfenden et al., 2012). Good practices using autistic adult-specific long-term treatments, however, are mostly unexplored. The Autism Spectrum Disorders in the European Union (ASDEU) project conducted a survey to collect information on services availability and experience related to autistic adult interventions in 11 European countries with the overall aim of determining how services for interventions were delivered to autistic adults. In particular, the present project aimed to investigate how well or how widely recommendations for autistic adults’ interventions were implemented in practice in community settings. Survey respondents were autistic adults, carers of autistic adults, and professionals in adult services. Questions concerned recommended items to consider when deciding on an intervention for autistic adults, factors of positive intervention outcomes and factors to be considered when deciding on an intervention for challenging behavior such as self-harm or injury to others. In addition, the survey explored the use of psychosocial and pharmacological interventions, and family interventions for members of an adult’s family.

In adulthood, some of the most relevant challenges that prompt interventions arise from core symptoms (i.e., repetitive/restricted behaviors; deficits in social skills) and poor adaptive functioning that may impede independent living, attendance at university/college and employment (Matson et al., 2016; Ratto & Mesibov, 2015). Specific psychosocial interventions have been developed for autistic adults targeting communication, social interaction and flexible thinking and behavior (e.g., social skills training; applied behavior analysis) (Matson et al., 1996; Odom et al., 2010). Other examples of promising training areas are vocational training (e.g., interview skills; supported employment; Morgan et al., 2014; Nicholas et al., 2015), anxiety management training (e.g., cognitive behavioral therapy; Lang et al., 2010), self-management techniques, video modeling, chaining, and individual work systems (Hume et al., 2009).

Autistic adult-specific guidelines, policies and services on interventions must be based on solid evidences (Nicholas et al., 2017). Efforts in this direction have been provided online by the National Institute for Health and Clinical Excellence (NICE, 2012). Autism Europe (2013) and the National Audit Office (2009) are other European examples of freely available quality standards for autistic adults’ services. Despite establishment of the foregoing guidelines on intervention for autistic adults, there is a lack of knowledge on how and which interventions for autistic adults are provided within the local communities. Thus, the present work aimed to collect experiences and perceptions on local interventions services’ use directly from autistic adults, carers and professionals to identify intervention practice gaps and chances for improvement.

## Methods

### Survey Description

The survey questions were created from a variety of published guidelines and recommendations regarding interventions for autistic adults (i.e., Autism Europe, 2013; Kendall et al., 2013; National Audit Office, 2009; NICE, 2012; Think Autism: Updating the 2010 Adult Autism Strategy). The survey questions and response options are presented in the Supplementary Material 1. The answer choices were designed to gauge how closely the respondent’s experiences with local intervention services matched the published recommendations. Three versions of the survey were developed to target autistic adults; family/caregivers of autistic adults (NOT necessarily the carers of the adults who participated in this study themselves); and administrators/professionals/service providers for adults. The draft surveys went through several stages of revision with inputs from the experts in all ASDEU sites. An autistic adult tested the on-line version for autistic adults and gave feedback.

Responders were instructed to select answers that seemed to suit most closely with what they knew or had experienced and to answer to the best of their knowledge and experience. Questions were written using everyday language and avoiding technical terms that might not be understood or applicable across different countries. The present study used data from two sections of the survey: (1) demographic characteristics of responders, including 12 questions for the autistic adults, 9 for carers, and 7 for professionals; (2) intervention practices for autistic adults, including 19 questions for autistic adults, 15 for carers, and 9 for professionals. The interventions section was restricted to responders who had recent adult intervention experience, e.g., autistic adults who had experience in the last two years with an intervention.

### Recruitment and Survey Distribution

The lead site for the adult services component of ASDEU (Denmark) provided all ASDEU partners with information and suggestions on how and to whom surveys could be distributed. Subsequently, all partners sent out survey notices and invitations to participate to autism organizations (national, local, voluntary) and service providers organizations (public and private, including residential facilities, job training and education programs). Furthermore, these organizations were encouraged to publish the survey links through their channels (e-newsletters, websites, or social media accounts). The researchers at each site also disseminated their surveys through their professional networks and on social media.

The survey was carried out over 10.5 months in 2017. It was launched in mid-February 2017 in three languages (English, Spanish, and Danish). By mid-September 2017, all three versions of the survey had been launched in 11 languages (English, Spanish, Danish, French, Polish, Icelandic, German, Finnish, Italian, and Romanian, as well as Portuguese for the professional version); data for this analysis were based on the total responses obtained up to December 2017.

Each ASDEU site obtained local ethical approval as needed before distributing the survey in their country. All procedures in studies involving human participants were in accordance with the ethical standards of the institutional and/or national research committee and with the World Medical Association Declaration of Helsinki and its later amendments or comparable ethical standards. Prior to starting the survey, responders had to read the information about the survey and give their informed consent electronically. No personal identifying information was collected. For analysis, data were handled in aggregated form; no feedback to participants was provided nor were individual respondent’s results reported. The background information section of the survey obtained a few demographic characteristics in order to classify the respondent for analysis purposes (e.g., gender, age, highest education level, country of residence, population size of the community where living/working).

### Analysis Methods

Overall, data from 2009 completed or partially completed surveys were distributed as follows: autistic adults (n = 667), carers of autistic adults (n = 591) and professionals (n = 751). For the purpose of the present study, only demographic characteristics and responses specific to intervention for autistic adults were analyzed. Only respondents who reported that they had recent knowledge of or experience with a particular intervention service were eligible to answer the relevant questions; all other respondents were automatically skipped to the next question. Thus, the intervention section was completed by a different number of responders depending on the specific question.

We analyzed the demographic characteristics and responses of 697 responders who were eligible to answer the intervention section (some variation in sample size per question depending on the intervention). These 697 responders answered ‘Yes’ to the following eligibility questions: ‘This section should be answered ONLY if you are in an intervention now or in the last two years, such as individual or group therapy to improve life skills or taking medicine for depression’ (autistic adult, 38%, n = 263); ‘You should answer this section ONLY if you have experience in the last two years with an intervention for the autistic adult. For example, the intervention could be individual or group therapy to improve life skills or taking medicine for depression’ (carer, 43%, n = 302); ‘Do you have knowledge of and current work experience (in the last two years) in interventions, such as individual or group therapy or medication, for adults on the autism spectrum?’ (professional, 19%, n = 132). Aggregated descriptive statistics by respondent group were calculated for all questions. We performed stratified analysis to see if variation in responses about intervention types was associated with the autistic adults’ level of independence/support needs reported by carers (Supplementary Material 8).

## Results

### Demographics

The 697 responders were mostly women (autistic adults: 68%, n = 180; carers: 85%, n = 256; professionals: 78%, n = 103), while the autistic adults cared for by carers were mainly men (71%, n = 215). Almost half of the autistic adults (40%) were over 35 years of age whereas only 17% of the carers’ adults were over 35. Participants were primarily living in Denmark (26%, n = 198), France (17%, n = 113), Finland (17%, n = 105), Spain (13%, n = 95), Poland (9%, n = 62), Italy (9%, n = 56), and Iceland (4%, n = 32) and lived in cities that are not capital cities (69%; n = 485). Most of the autistic adult responders reported to be currently in college/university education program (17%, n = 44) or had completed study at a college/university level (27%, n = 70). Over half (57%, n = 149) of the autistic adult responders were unemployed, and the most common reason for unemployment was having a disability that prevents them from having a job (40%, n = 59). One quarter (24%) of the autistic responders was diagnosed between 16 and 25 years old, while the rest were 26 years of age or older (Supplementary Material 2).

About half of autistic adults cared by carers had some level of independence (high level of independence, 9%, n = 26; some independence but need support, 40%, n = 122), whereas the other half required a high level of support (needs a high level of support in daily living, 36%, n = 110; needs high level institution-like care, 15%, n = 44). Sixty percent of the autistic adults cared by carers were diagnosed between 16 and 25 years old, while the rest were 26 years of age or older (Supplementary Material 2).

The most commonly represented professional backgrounds were psychologists (46%, n = 61), teachers/pedagogues (13%, n = 17), and psychiatrists (11%, n = 14) (Supplementary Material 2).

### Alignment with Guidelines: Recommendations Regarding Practices Around Interventions for Autistic Adults

#### Recommended Considerations When Deciding on an Intervention

As shown in Supplementary Material 3, among all three groups, more than 50% experienced each of 12 recommended items to consider when deciding on an intervention (i.e., age, history of previous interventions; other kinds of interventions had in the past; experience in past interventions; presence of intellectual impairment (by the carers and professionals); presence of other chronic conditions; presence of things that are part of the problem; what is needed to implement the intervention; if the adult will be able to accept the intervention; the level of the adult’s motivation; adult’s level of stress and well-being; how well the intervention might work (by the adults and carers). In contrast, consideration of gender when deciding on an intervention was experienced by less than a half of adults or carers, and considered standard/routine practice by only 52% of professionals. Furthermore, less than the 37% of the carers experienced the consideration of whether the adult asked for the intervention, or if the adult was asked to give consent (Supplementary Material 3).

When totaling the number of recommended features (when deciding on an intervention) that each participant experienced, most autistic adults, carers, and professionals experienced the majority of the recommended items. Among the autistic adults, 59% (n = 105) experienced eight or more of the 14 recommended features. Among carers, 51% (n = 94) experienced 10 or more of the 15 recommended features. And among professionals, 56% (n = 72) experienced all 13 recommended features (Supplementary Material 4).

#### Recommended Factors as Part of the Intervention Plan and Implementation

Adults and carers were asked if, after the intervention started, there was a regular review to check improvement made by the adult and adult’s difficulties with intervention. As shown in Supplementary Material 5, more than a half of the adults and carers experienced these two recommended features. Professionals were asked about four recommended features (i.e., written protocol for implementing intervention; monitoring and recording of adverse events; monitoring adherence; regular review for improvements/challenges). More than the 65% of professionals reported that each of these four recommended features were standard routine practice/often considered, although more than the 12% of professionals rarely or never considered each of these recommended factors as part of the intervention plan for an autistic adult.

### Uses of Psychosocial Interventions

**Fig. 1:**
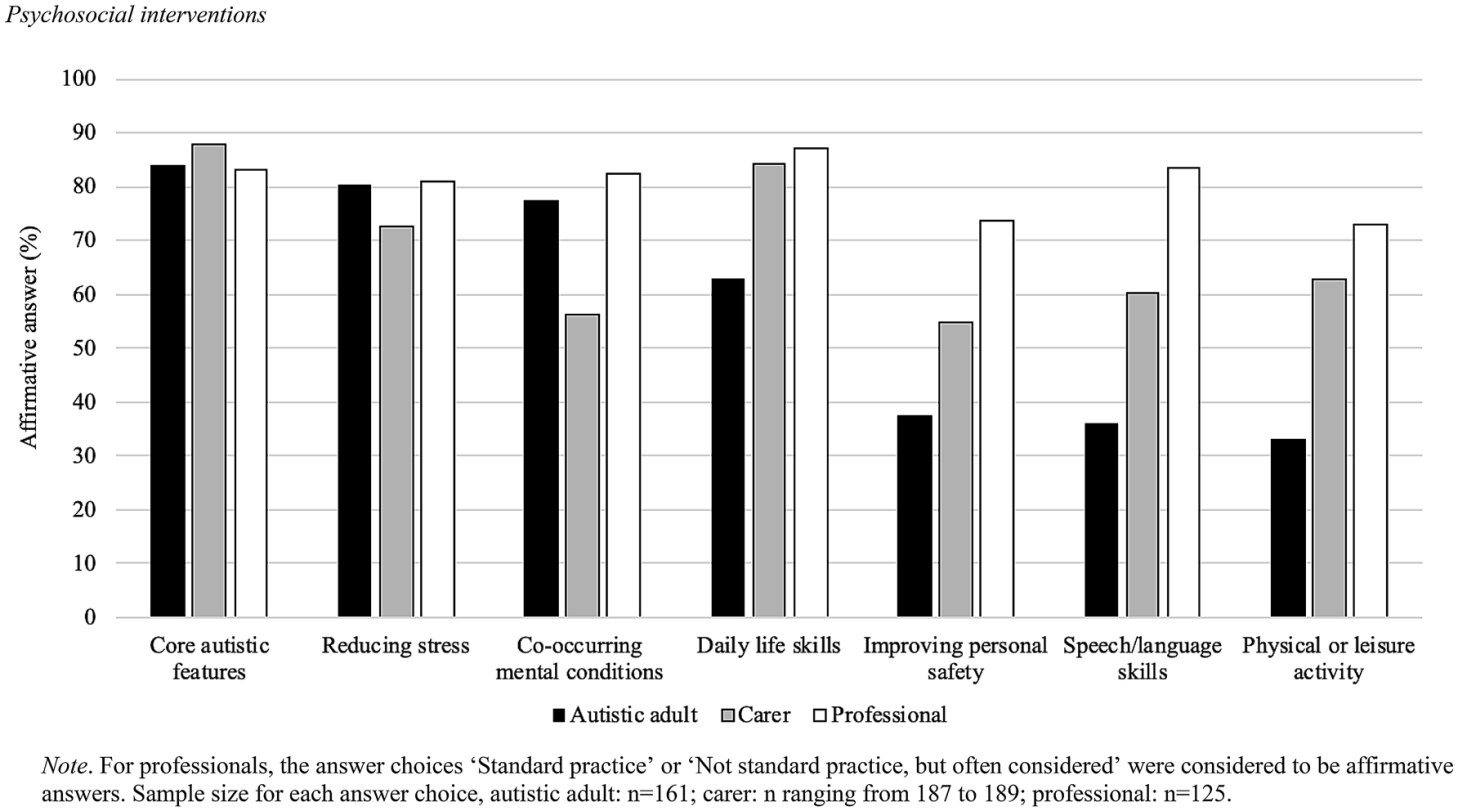
Psychosocial interventions. *Note*: For professionals, the answer choices ‘Standard practice’ or ‘Not standard practice, but often considered’ were considered to be affirmative answers. Sample size for each answer choice, autistic adult: n = 161; carer: n ranging from 187 to 189; professional: n = 125

As shown in Table 1, among adults and carers, about 63% reported that the adult received—in the last two years—psychosocial interventions (e.g., individualized interventions; group therapy; support groups). More than the 53% of adults and carers reported that their psychosocial interventions were for core autistic features, daily life skills, co-occurring mental conditions or reducing stress. A discrepancy was observed between adults (< 32%) and carers (> 49%) in reporting psychosocial interventions used for improving personal safety, speech/language skills, and physical or leisure activity (Fig. 1). More than 73% of professionals reported that psychosocial interventions were standard routine practice/often considered for all features (i.e., core autistic features; reducing stress; co-occurring mental conditions; daily life skills; improving personal safety; speech/language skills; physical or leisure activity) (Fig. 1).

**Table 1:** Psychosocial interventions

	Autistic adult				Carer				Professional					
	Yes	No	Do not know	N	Yes	No	Do not know	N	Standard/routine practice	Not standard but often considered	Rarely considered	Never considered	Do not know	N
Adult received a psychosocial intervention in the last 2 years	161 (63.1)	85 (33.3)	9 (3.5)	255	189 (63.4)	102 (34.2)	7 (2.4)	298	N/A					
Core autistic features	131 (81.4)	25 (15.5)	5 (3.1)	161	158 (84.0)	22 (11.7)	8 (4.3)	188	75 (60.0)	29 (23.2)	9 (7.2)	1 (0.8)	11 (8.8)	125
Reducing stress	121 (72.5)	30 (18.6)	10 (6.2)	161	129 (68.3)	49 (25.9)	11 (5.8)	189	69 (55.2)	32 (25.6)	11 (8.8)	5 (4.0)	8 (6.4)	125
Co-occurring mental conditions	122 (75.8)	36 (22.4)	3 (1.9)	161	100 (53.2)	78 (41.5)	10 (5.3)	188	62 (49.6)	41 (32.8)	11 (8.8)	3 (2.4)	8 (6.4)	125
Daily life skills	98 (60.9)	58 (36.0)	5 (3.1)	161	154 (81.5)	29 (15.3)	6 (3.2)	189	81 (64.8)	28 (22.4)	9 (7.2)	0	7 (5.6)	125
Improving personal safety	58 (36.0)	98 (60.9)	5 (3.1)	161	92 (48.7)	76 (40.2)	21 (11.1)	189	56 (44.8)	36 (28.8)	13 (10.4)	6 (4.8)	14 (11.2)	125
Speech/language skills	56 (34.8)	100 (62.1)	5 (3.1)	161	107 (57.2)	71 (38.0)	9 (4.8)	187	66 (52.8)	38 (30.4)	10 (8.0)	1 (0.8)	10 (8.0)	125
Physical or leisure activity	51 (31.7)	103 (64.0)	7 (4.4)	161	113 (60.1)	67 (35.6)	8 (4.3)	188	50 (40.0)	41 (32.8)	20 (16.0)	2 (1.6)	12 (9.6)	125

Values expressed as number of responders and frequencies (in parenthesis). N/A = Question not available for the correspondent group. The first question was the following: autistic adult: *‘Were you* (for carer: *Has the adult received*) *in a psychosocial intervention in the last 2 years? A psychosocial intervention can be something like individual or group therapy or a support group, but medicines and medical procedures are not part of it.’;* The questions on the other characteristics were the following: autistic adult and carer: ‘*If yes, was the psychosocial intervention for:…’*; Professional: ‘*In this question we are asking about psychosocial interventions (e.g., individualized interventions, group therapy, support groups). Based on your knowledge and work experience in the "area where you work now", how often are psychosocial interventions with autistic adults used for:…’*

### Uses of Pharmacological Interventions

Over half (57%, n = 146) of autistic adults and 42% (n = 124) of carers reported that the adults received an intervention in the last 2 years that only used medicines or medical procedures. The majority (62%) of the carers of high-level support or institution-like care autistic adults received only pharmacological intervention (Supplementary material 8). Among these responders, 52% to 85% used medicines or medical procedures for helping to control sleep problems, moods or emotions, or treating mental conditions. More than the 83% of the professionals reported that pharmacological interventions were standard routine practice/often considered for helping to control these problems (Table 2).

**Table 2:** Pharmacological interventions

	Autistic adult				Carer				Professional					
	Yes	No	Do not know	N	Yes	No	Do not know	N	Standard/routine practice	Not standard but often considered	Rarely considered	Never considered	Do not know	N
*Pharmacological intervention*														
Adult received only pharmacological intervention in the last 2 years	146 (57.3)	103 (40.4)	6 (2.4)	255	124 (42.0)	164 (55.6)	7 (2.4)	295	N/A					
Treating mental conditions, such as depression	124 (84.9)	19 (13.0)	3 (2.1)	146	76 (62.3)	41 (33.6)	5 (4.1)	122	60 (48.4)	48 (38.7)	4 (3.2)	2 (1.6)	10 (8.1)	124
Helping to control sleep problems	81 (55.5)	60 (41.1)	5 (3.4)	146	63 (51.6)	55 (45.1)	4 (3.3)	122	49 (39.5)	54 (43.5)	9 (7.3)	1 (0.8)	11 (8.9)	124
Helping to control moods or emotions (dysregulation)	72 (49.3)	64 (43.8)	10 (6.9)	146	82 (66.7)	38 (30.9)	3 (2.4)	123	58 (46.8)	50 (40.3)	7 (5.6)	1 (0.8)	8 (6.5)	124
*NOT recommended pharmacological intervention for core autistic symptoms*														
Adult received only pharmacological intervention for treating core autism spectrum symptoms in the last 2 years	36 (14.1)	200 (78.4)	19 (7.5)	255	60 (20.4)	220 (74.8)	14 (4.8)	294	N/A					
Antidepressants	26 (72.2)	9 (25.0)	1 (2.8)	36	28 (47.5)	30 (50.8)	1 (1.7)	59	33 (26.8)	53 (43.1)	13 (10.6)	3 (2.4)	21 (17.1)	123
Antipsychotics	N/A				34 (56.7)	24 (40.0)	2 (3.3)	60	43 (35.0)	36 (29.3)	16 (13.0)	2 (1.6)	26 (21.1)	123
Seizure medications	8 (22.2)	27 (75.0)	1 (2.8)	36	12 (20.3)	45 (76.3)	2 (3.4)	59	40 (32.5)	29 (23.6)	18 (14.6)	3 (2.4)	33 (26.8)	123
Special diets	6 (16.7)	30 (83.3)	0	36	16 (27.1)	43 (72.9)	0	59	11 (8.9)	21 (17.1)	43 (35.0)	23 (18.7)	25 (20.3)	123
Oxytocin	1 (2.8)	32 (88.9)	3 (8.3)	36	2 (3.4)	51 (86.4)	6 (10.2)	59	1 (0.8)	2 (1.6)	18 (14.6)	48 (39.0)	54 (43.9)	123
Secretin	1 (2.8)	34 (94.4)	1 (2.8)	36	0	51 (86.4)	8 (13.6)	59	0	1 (0.8)	12 (9.8)	51 (41.5)	59 (48.0)	123
Chelation	0	36 (100.0)	0	36	1 (1.7)	57 (96.6)	1 (1.7)	59	0	2 (1.6)	22 (17.9)	56 (45.5)	43 (35.0)	123
Hyperbaric oxygen therapy	0	35 (97.2)	1 (2.8)	36	1 (1.7)	52 (88.1)	6 (10.2)	59	1 (0.8)	0	16 (13.0)	53 (43.1)	53 (43.1)	123
Testosterone	0	35 (97.2)	1 (2.8)	36	0	53 (89.8)	6 (10.2)	59	1 (.8)	2 (1.6)	16 (13.0)	47 (38.2)	57 (46.3)	123
Stimulant	N/A				N/A				11 (8.9)	22 (17.9)	32 (26.0)	15 (12.2)	43 (35.0)	123

Values expressed as number of responders and frequencies (in parenthesis). N/A = Question not available for the correspondent group. The questions on pharmacological interventions were the following: autistic adult and carer: ‘*Were you in* (for carer: *has the adult received*) *an intervention in the last 2 years that only used medicines and no other kinds of therapy?’; ‘If yes, was the medicine used for:…’;* Professionals: *‘In this question we are asking about pharmacological interventions. Based on your knowledge and work experience in the "area where you work now", how often are pharmacological interventions for autistic adults considered for:…’.* The questions on NOT recommended pharmacological intervention for core autistic symptoms were, for autistic adult and carer: ‘*Were you in (*for carer: *Has the adult received) an intervention in the last 2 years for treating core autism spectrum symptoms, such as social difficulties, that only used medicines or medical procedures?’; ‘If yes, did the medicines or medical procedures include:…’*; Professional: *‘In this question we ask about either pharmacological or medical-type interventions used for core autism spectrum behaviors. Based on your knowledge and work experience in the "area where you work now", how often are the following pharmacological or medical-type interventions used for core autism spectrum behaviors specifically:…’*

### Alignment with Guidelines: Recommendations Advising Against Uses of Pharmacological Interventions for Core Autistic Symptoms

Table 2 shows that 14% (n = 36) of autistic adults and 20% (n = 60) of carers reported that the adults received pharmacological interventions NOT recommended for treating core autistic symptoms in the last 2 years. The majority (63%) of the carers of high-level support or institution-like care autistic adults received only pharmacological intervention for treating core autism spectrum symptoms (Supplementary material 8). Among those responders that reported pharmacological interventions for treating core autism spectrum symptoms, the most frequently reported medicines by autistic adults were antidepressants (72%). Among carers 57% reported use of antipsychotics. More than 64% of professionals reported that antidepressants and antipsychotics were standard routine practice or often considered for core autism spectrum behaviors.

About 17% to 27% of autistic adults and carers reported that seizure medications and special diets were considered for core autism spectrum symptoms. Whereas, more professionals reported seizure medications (56%), special diets (26%), or stimulants (27%) as a standard routine practice or often considered for core autism spectrum behaviors. Notably, across all medicines or medical procedures, a high frequency of professionals (17% to 48%) reported that they did not know how often the pharmacological or medical-type intervention was used for core autism spectrum behaviors. Small fractions (0% to 3%) among all groups reported the use of oxytocin, secretin, chelation, hyperbaric oxygen therapy, or testosterone for treating core autistic symptoms.

Among the autistic adults (86%, n = 209) and carers (80%, n = 235), most did not experience any of the NOT recommended pharmacological intervention for core autistic symptoms in adulthood. Among professionals, 69% (n = 85) experienced three or less of the NOT recommended features (Supplementary Material 6).

### Types of Family Interventions for Members of Adult’s Family

As shown in Table 3, less than 9% of the autistic adults and less than 22% of carers reported that a family member, sibling, partner, or carer received at least one intervention in the last 2 years. For example, less than the 3% of carers reported experiencing marital counselling. In contrast, over half of professionals reported family interventions as standard routine practice or often considered for families, siblings, partners, or carers of autistic adults, except for marital counselling or respite care. It is worth noticing that marital counseling was reported more often by carers of high or some independence autistic adults (73%), while respite care was reported more often by carers of high-level support or institution-like care autistic adults (75%) (Supplementary Material 8).

**Table 3:** Family interventions

	Autistic adult (N = 484)			Carer (N = 449)			Professional (N = 123)				
	Yes	No	Do not know	Yes	No	Do not know	Standard/routine practice	Not standard but often considered	Rarely considered	Never considered	Do not know
Individual therapy	44 (9.1)	413 (85.3)	27 (5.6)	63 (14.0)	366 (81.5)	20 (4.5)	38 (30.9)	38 (30.9)	23 (18.7)	11 (8.9)	13 (10.6)
Care planning for the autistic adult	N/A			N/A			53 (43.1)	31 (25.2)	27 (22.0)	4 (4.1)	8 (6.5)
Training on autism spectrum and care planning	35 (7.7)	420 (86.8)	29 (6.0)	98 (21.8)	331 (73.7)	20 (4.5)	44 (35.8)	36 (29.3)	21 (17.1)	7 (5.7)	15 (12.2)
Support groups	33 (6.8)	425 (87.8)	26 (5.4)	75 (16.7)	357 (79.5)	17 (3.8)	34 (27.6)	45 (36.6)	29 (23.6)	10 (8.1)	5 (4.1)
Advice about care for a person on the autism spectrum	N/A			N/A			57 (46.3)	35 (28.5)	23 (18.7)	1 (0.8)	7 (5.7)
Marital counseling	26 (5.4)	438 (90.5)	20 (4.1)	15 (3.3)	414 (92.2)	20 (4.5)	7 (5.7)	25 (20.3)	31 (25.2)	34 (27.6)	26 (21.1)
Respite care	17 (3.5)	444 (91.7)	23 (4.8)	87 (19.4)	347 (77.3)	15 (3.3)	19 (15.5)	35 (28.5)	37 (30.1)	15 (12.2)	17 (13.8)

Values expressed as number of responders and frequencies (in parenthesis). N/A = Question not available for the correspondent group. The questions were the following: autistic adult: *‘In the last 2 years, has anyone close to you been in any of the following interventions for family members, siblings, partners or carers of autistic adults? These kinds of interventions help people to better understand autism spectrum and how to better support the autistic adult and themselves.’;* carer*: ‘In the last 2 years, has anyone close to the adult like a family member, sibling, partner or carer received any of the following interventions? These kinds of interventions help people to better understand autism spectrum and how to better support the autistic adult and themselves’;* professional: *‘Based on your knowledge and work experience in the "area where you work now", how often are the following interventions available for families, siblings, partners or carers of autistic adults?’*

### Challenging Behavior (Self-Harm or Injury to Others)

Table 4 shows that 38% of autistic adults reported self-harm (such as hitting themselves) or trying to harm themselves (including trying to commit suicide), and 12% reported harming other people (such as hitting other people) in the last two years. Almost half (46%) of carers reported that the adult they cared for had challenging behavior, for example self-harm, attempted suicide, or aggression towards others in the last two years. Among responders who reported having experienced challenging behaviors, less than 26% of autistic adults and 55% of carers reported to have been in an intervention to help change the behavior.

**Table 4:** Self-harm or harm to other people interventions

	Autistic adult *Self-harm / Harm to other*^a^				Carer				Professional					
	Yes	No	Do not know	N	Yes	No	Do not know	N	Standard/routine practice	Not standard but often considered	Rarely considered	Never considered	Do not know	N
Adult had challenging behaviors in the last 2 years	181 (37.6) / 58 (12.1)	284 (58.9) / 414 (86.1)	17 (3.5) / 9 (1.9)	482 / 481	207 (46.2)	232 (51.8)	9 (2.0)	448	N/A					
Adult had an intervention to change the behavior in the last 2 years	39 (21.6) / 15 (25.9)	133 (73.5) / 37 (63.8)	9 (4.9) / 6 (10.3)	181 / 58	113 (54.6)	88 (42.5)	6 (2.9)	207	N/A					
*Intervention type*														
Psychosocial interventions, only	23 (60.5) / 9 (60.0)	15 (39.5) / 6 (40.0)	0 / 0	38 / 15	43 (38.4)	65 (58.0)	4 (3.6)	112	39 (32.8)	42 (35.3)	18 (15.1)	4 (3.4)	16 (13.5)	119
Psychosocial + pharmacological interventions	18 (47.4) / 8 (53.3)	18 (47.4) / 7 (46.7)	2 (5.3) /	38 / 15	56 (50.0)	55 (49.1)	1 (0.9)	112	55 (46.2)	40 (33.6)	9 (7.6)	2 (1.7)	13 (10.9)	119
Pharmacological interventions, only	9 (23.7) / 7 (46.7)	29 (76.3) / 8 (53.3)	0 / 0	38 / 15	37 (33.0)	74 (66.1)	1 (0.9)	112	12 (10.1)	35 (29.4)	29 (24.4)	20 (16.8)	23 (19.3)	119
*Recommended factors to be considered when deciding on an intervention for challenging behavior*														
Presence of stressful situation	35 (92.1) / 11 (73.3)	2 (5.3) / 3 (20.0)	1 (2.6) / 1 (6.7)	38 / 15	82 (72.6)	25 (22.1)	6 (5.3)	113	85 (71.4)	24 (20.2)	6 (5.0)	1 (0.8)	3 (2.5)	119
High levels of anxiety	34 (89.5) / 11 (73.3)	3 (7.9) / 2 (13.3)	1 (2.6) / 2 (13.3)	38 / 15	74 (65.5)	33 (29.2)	6 (5.3)	113	78 (65.6)	34 (28.6)	3 (2.5)	1 (0.8)	3 (2.5)	119
Communication problems	33 (86.8) / 13 (86.7)	5 (13.2) / 2 (13.3)	0 / 0	38 / 15	86 (76.1)	24 (21.1)	3 (2.7)	113	87 (73.1)	20 (16.8)	7 (5.9)	2 (1.7)	3 (2.5)	119
Difficulties in personal relations	32 (84.2) / 9 (60.0)	6 (15.8) / 5 (33.3)	0 / 1 (6.7)	38 / 15	68 (60.2)	42 (37.2)	3 (2.6)	113	80 (67.2)	24 (20.2)	8 (6.7)	1 (0.8)	6 (5.0)	119
Presence of a mental disorder	30 (78.9) / 8 (53.3)	8 (21.1) / 6 (40.0)	0 / 1 (6.7)	38 / 15	70 (61.9)	35 (30.9)	8 (7.1)	113	80 (67.2)	25 (21.0)	10 (8.4)	0	4 (3.4)	119
Challenges in the physical environment	29 (76.3) / 9 (60.0)	8 (21.1) / 6 (40.0)	1 (2.6) / 0	38 / 15	69 (61.1)	36 (31.9)	8 (7.1)	113	74 (62.2)	31 (26.1)	10 (8.4)	1 (0.8)	3 (2.5)	119
Things reinforcing the challenging behavior	28 (73.7) / 9 (60.0)	8 (21.1) / 4 (26.7)	2 (5.3) / 2 (13.3)	38 / 15	78 (69.0)	25 (22.1)	10 (8.9)	113	83 (69.8)	22 (18.5)	8 (6.7)	1 (0.8)	5 (4.2)	119
Recent changes to routine	25 (65.8) / 11 (73.3)	13 (34.2) / 4 (26.7)	0 / 0	38 / 15	74 (65.5)	35 (31.0)	4 (3.5)	113	83 (69.8)	21 (17.7)	8 (6.7)	3 (2.5)	4 (3.4)	119
Patterns in the challenging behavior	N/A				72 (63.7)	30 (26.6)	11 (9.7)	113	77 (64.7)	24 (20.2)	9 (7.6)	1 (0.8)	8 (6.7)	119
Recent changes in personal circumstances	20 (52.6) / 5 (33.3)	17 (44.7) / 9 (60.0)	1 (2.6) / 1 (6.7)	38 / 15	43 (38.1)	66 (58.4)	4 (3.5)	113	85 (71.4)	20 (16.8)	8 (6.7)	2 (1.7)	4 (3.4)	119
Physical disorder	11 (28.9) / 3 (20.0)	23 (60.5) /11 (73.3)	4 (10.5) /1 (6.7)	38 / 15	54 (47.8)	51 (45.1)	8 (7.1)	113	79 (66.4)	26 (21.9)	7 (5.9)	0	7 (5.9)	119

^a^Self-harm or harm to other people intervention questions for autistic adults were in separate sections. Therefore, the first value in each cell below corresponds to answers to the self-harm questions and the second value corresponds to answers to the harm to others questions.

*Note*. Values expressed as number of responders and frequencies (in parenthesis). N/A = Question not available for the correspondent group. The first question was the following: autistic adult: *‘In the last 2 years have you had any behavior where you were harming yourself (such as hitting yourself) or trying to harm yourself (including trying to commit suicide)?’ / In the last 2 years have you had any behavior where you were harming other people (such as hitting other people)?’;* carer*: ‘In the last 2 years has the adult had any challenging behavior, for example self-harm, attempted suicide or aggression towards others?’.* The second question was the following: autistic adult and carer: ‘*If yes, were you* (for carer: *Has the adult been*) *in an intervention to help change the behavior?*’. Intervention type questions were the following: autistic adult and carer: *‘Were* (for carer: *Has the adult been*) *you in any of the following types of interventions in the last 2 years to help change the behavior?’*; professional*: ‘Based on your knowledge and work experience in the "area where you work now", how often are the following types of interventions used for challenging behavior in autistic adults:…’.* Recommended factors to be considered when deciding on an intervention for challenging behavior questions were the following: autistic adult and carer: *‘If yes, were any of the following factors talked about* (for adult: *with you*) *when deciding on an approach to help change the behavior?’*; professional: *‘Based on your knowledge and work experience in the "area where you work now", if an adult on the autism spectrum displays challenging behavior (e.g., self-harm or aggression) how often are the following features considered when deciding on an approach to change the behavior?’*

#### Types of Interventions Used for Challenging Behavior (Self-Harm or Injury to Others)

Among autistic adults, psychosocial interventions were the most commonly experienced interventions for challenging behavior (for self-harm: 61%; for injury to others: 60%), followed by combined psychosocial and pharmacologic intervention (for self-harm: 47%; for injury to others: 53%). Combined psychosocial and pharmacological interventions were reported to be used for challenging behavior by half of carers, whereas 38% of carers reported psychosocial interventions, and 33% pharmacological interventions only. Combined psychosocial and pharmacological interventions, and pharmacological interventions only were reported by more than the 61% of the carers of high-level support or institution-like care (Supplementary Material 8). Among professionals, 68% reported that psychosocial interventions, and 80% that combined psychosocial and pharmacologic interventions were standard routine practice or often considered for treating challenging behavior. Pharmacological treatments were reported to be standard routine practice or often considered by 40% of the professionals (Table 4).

#### Recommendations for Factors to be Considered When Deciding on an Intervention for Challenging Behavior (Self-Harm or Injury to Others)

Among all groups, more than half experienced each of the majority of the recommended factors to be considered when deciding on an intervention for challenging behavior (i.e., difficulties in personal relations, challenges in the physical environment, communication problems, presence of a mental disorder, recent changes to routine, things reinforcing the challenging behavior, high levels of anxiety, presence of stressful situation, patterns in the challenging behavior (by the carers and professionals). Whereas, the recommended factors experienced by less than the 50% of the autistic adults or carers were the following: recent changes in personal circumstances and presence of a physical disorder. In contrast, 88% of the professionals reported these two recommended factors were often considered or as a standard routine practice (Table 4).

In total, most autistic adults, carers and professionals experienced the majority of the recommended factors to be considered when deciding on an intervention for challenging behavior. Among the autistic adults, 53% (n = 20) experienced 8 or more of the 10 recommended features. Among carers, 67% (n = 10) experienced 6 or more of the 9 recommended features, and among professionals, 71% (n = 84) experienced all eleven recommended features (Supplementary Material 7).

## Discussion

A sample of 697 autistic adults, carers and professionals from eleven European countries responded to the ASDEU on-line survey on intervention services. Their responses indicate a fairly high level of concordance between many, but not all, recommended features of intervention services for autistic adults and carers’ current experiences as well as insight into professionals’ knowledge of and perceptions of autistic adult intervention services.

### Alignment with Guidelines: Recommendations Regarding Practices Around Interventions for Autistic Adults

#### Recommended Considerations When Deciding on an Intervention

It is very important when planning interventions to assess factors that are part of the problem and, to achieve optimal outcome, to tailor the intervention to the autistic adults and their carers characteristics: motivation, other interventions in the past, expectations for the actual intervention, preference, consent, and gender. Most of the recommended considerations when deciding on an intervention for an autistic adult were experienced by the at least 50% of the participants. Notable exceptions to this pattern, however, concerned considerations of gender, if the adult asked for the intervention, and if the adult was asked to give consent which were experienced by a minority of carers. Professionals are advised to take into consideration the adult's gender when deciding on an intervention because females may display a different phenotype or different patterns of stereotyped behaviors (Kirkovski et al., 2013; Van Wijngaarden-Cremers et al., 2014) compared to males, with more socially appropriate interests, in line with the camouflage theory (Mattila et al., 2010). In the present study, gender may have not been considered by most professionals because there are no gender-based targeted interventions. In addition, the majority of prior intervention studies were conducted on males (Bishop et al., 2013; Cappadocia & Weiss, 2011; Ke et al., 2017; Roth et al., 2013; Spain & Blainey, 2015) and the outcomes of interventions for female autistic adults is still unclear. This gap should be mitigated by implementing research depicting interventions’ outcomes depending on the gender of the autistic adults and directions towards specific interventions gender targeted.

In addition, in the present study, carers infrequently reported the consideration of autistic adult’s preferences and consent when planning for an intervention, which may relect the fact that half of the carers cared for adults needing a high-level of support or institution-like care. The direct involvement of the autistic adult in the intervention by asking their preference and consent, however, is not only ethical practice, but may facilitate an effective engagement to the treatment (Entwistle, & Watt, 2006). The will of autistic adults, including those who require a high level of support, must be heard.

#### Recommended Factors as Part of the Intervention Plan and Implementation

More than a half of responders experienced the recommended factors as part of the intervention plan and implementation. However, there is still room to improve rates of adherence to all recommended items since more than 28% of autistic adults and carers never experienced the recommendations to conduct checks for improvement made by the adult and the adult’s difficulties with the intervention. Fully 12% of professionals rarely or never considered in their standard practice a regular review for improvements or challenges, a written protocol for implementing intervention, monitoring adherence to the intervention, or monitoring and recording of adverse events as part of the intervention plan and implementation. Monitoring the course of the intervention, improvements, challenges and adherence may be crucial to modify the intervention strategies applied and improving positive outcomes.

### Uses of Psychosocial Interventions

The majority of the autistic adults received, in the last two years, a psychosocial intervention (e.g., individualized interventions; group therapy; support groups). As expected and recommended by the NICE guidelines, psychosocial interventions were used for a wide array of behavioral and daily life issues. More than a half of the responders experienced psychosocial interventions for core autistic features, daily life skills, co-occurring mental conditions, or reducing stress. Not many (< 32%) autistic adults reported psychosocial interventions for speech/language skills, physical or leisure activity, or improving personal safety. However, more than the 70% of the professionals reported that psychosocial interventions were used for all these features as standard routine practice or often considered.

The latter results from the autistic adults may reflect their relatively high levels of independence and functioning; it is possible that they did not need these types of support. Alternatively, these results may reflect discrepancies in the perceptions and experiences on local services’ use between adults and professionals. Previous studies have already revealed poor alignment between services recommendations and actual experiences by autistic adults (Crane et al., 2018; Mukaetova-Ladinska, & Stuart-Hamilton, 2016; Scattoni et al., 2021).

### Uses of Pharmacological Interventions

Many of the autistic adults (adults: 57%; adults’ carers: 42%) received a pharmacological intervention in the last two years. More than a half of these responders received pharmacological interventions to control sleep problems, moods/emotions or treating mental conditions. Whereas, a higher proportion of professionals (> 83%) answered that pharmacological interventions were standard routine practice or often considered for helping to control these problems. Again, discrepancies between autistic adults, carers, and professionals’ experiences and perceptions have been observed. However, the discrepancies have to be considered with caution. First, professionals were mainly non-medical specialists (e.g., psychologists; teachers/pedagogues) and also were answering in view of general services practice whereas the autistic adults and carers were reporting only their personal experiences. Further, autistic adults and carers may not have been able to properly distinguish the specific condition for which the pharmacological agents had been prescribed.

#### Alignment with Guidelines: Recommendations Advising Against Uses of Pharmacological Interventions for Core Autistic Symptoms

The NICE guidelines do NOT recommend the use of the following biomedical interventions for managing core symptoms of autistic adults: anticonvulsants, chelation, exclusion diets, vitamins, minerals, dietary supplements, drugs specifically designed to improve cognitive functioning, oxytocin, secretin, testosterone regulation, hyperbaric oxygen therapy, antipsychotic medication, and antidepressant medication (NICE, 2012). However, 14% of autistic adults and 20% of carers experienced only pharmacological interventions for treating core autism spectrum symptoms in the last two years, especially the use of antidepressants and antipsychotics. Again, the autistic adults and carers may not have been able to properly distinguish between uses of pharmacological agents and antidepressants specifically, for core autistic symptoms versus other problems, so these results should be viewed with caution. On the other hand, a larger proportion of professionals reported that antidepressants and antipsychotics were standard routine practice or often considered for core autism spectrum behaviors (for antidepressant: 70%; antipsychotics: 64%) and these results should also be viewed with caution. In view of these response rates, however, further study may be warranted to confirm or clarify the community rate of use of pharmacological interventions for core autism symptoms. Encouraging is the result that only a very small fraction (< 3%) of responders reported oxytocin, secretin, chelation, hyperbaric oxygen therapy, or testosterone being considered for treating core autistic symptoms.

### Types of Family Interventions for Members of Adult’s Family

The NICE guidelines recommend carers’ needs assessment and interventions for families, partners, and carers. Family interventions were relatively infrequently used by carers (< 22%), and such interventions were reported at even lower rates by adults (< 9%). These rates may reflect that, first, the autistic adults may not be aware of the interventions carried out by their family members, and second, the relatively high levels of independence and functioning of the autistic adults and perhaps less need for family support. Or, the results may reflect an unrecognized need. In contrast, more professionals (> 44%) reported family interventions as standard routine practice or often considered. It has to be recognized that autistic adults and carers’ perceptions were limited to their own personal experience while professionals’ perceptions were based on a large sample of professional experiences. Thus, autistic adults, carers, and professionals’ answers may not be directly comparable.

### Challenging Behavior (Self-Harm or Injury to Others)

A large minority (38%) of autistic adults reported self-harm behavior and 12% reported injury to others behavior in the last two years, while 46% of carers reported that the adult they cared for had these challenging behaviors in the last two years. Non-suicidal self-inflicted injuries and suicidal tendencies rates in autistic adolescents and adults are higher than non-autistic peers (Cassidy et al., 2018; Maddox et al., 2017), and more often is the cause of death (Schendel et al., 2016).

More that the 26% of autistic adults and 55% of carers experienced an intervention to help change challenging behaviors. Previous research highlighted the importance of the autistic adults and carers’ perception of receiving appropriate intervention which may impact their well-being (Burgess & Gutstein, 2007; Camm-Crosbie et al., 2019; Cassidy et al., 2018).

#### Types of Interventions Used for Challenging Behavior (Self-Harm or Injury to Others)

Psychosocial interventions for challenging behavior are recommended by the NICE guidelines. Antipsychotic medications in conjunction with psychosocial interventions for challenging behavior should be applicable when psychosocial or other interventions produce no or limited response (NICE, 2012). Psychosocial (> 60%) or combined psychosocial and pharmacologic (> 47%) strategies were the most common interventions for challenging behavior reported by adults. In contrast, 38% of carers reported psychosocial interventions for treating challenging behavior. The majority of professionals considered psychosocial interventions (68%) and the combined psychosocial and pharmacologic interventions (80%) as a standard routine practice or often considered for treating challenging behavior.

The NICE guidelines suggest that antipsychotic medication for challenging behavior on its own should be considered when psychosocial or other interventions cannot be delivered because of the severity of the challenging behavior. In our sample, pharmacological interventions for challenging behavior were reported least frequently by adults (self-harm: 24%; injury to others: 47%) and professionals (40%), and most frequently by carers (50%).

The carers reported the lowest rate of psychosocial intervention for challenging behavior among responders. These differences may reflect again different levels of independence and functioning of the autistic adult responders and the autistic adults cared for by the carers. However, the rates of pharmacological intervention for challenging behavior is still high among all participants. The present survey did not explore if the pharmacological intervention was used only when psychosocial or other interventions could not be delivered because of the severity of the challenging behavior, as recommended by the NICE guidelines. Future research should explore the rate and reasons behind use of pharmacological interventions for challenging behavior vis a vis the recommended clinical conditions.

### Recommendations for Factors to be Considered When Deciding Which Intervention to Use for Challenging Behavior (Self-Harm or Injury to Others)

The modalities and types of interventions for challenging behavior are also described in NICE guidelines. Before initiating interventions for challenging behavior, professionals should explore factors that may start or maintain the challenging behavior (i.e., care for physical disorders; treatment for any coexisting mental disorders; interventions already in place). The choice of the intervention should be personalized on the nature and severity of the behavior of the autistic adult, the person’s physical needs and capabilities, the physical and social environment, the capacity of professionals and carers to provide support, the adult’s and carer’s preferences, and the past history of care and support (NICE, 2012). Most of the recommendations for factors to be considered when planning an intervention for challenging behavior were experienced by the majority of adults, carers and professionals. The features least often experienced by adults or carers included recent changes in personal circumstances and the presence of a physical disorder. More than the 80% of professionals, however, reported that these two factors were standard routine practice or often considered.

Professionals should consider changes in personal circumstances and the presence of a physical disorder when deciding on an intervention for challenging behavior since they are important factors that may influence intervention outcomes (Kendall et al., 2013).

### Limitations

The present study has some limitations that should be considered when interpreting the results. First, the sample was not collected using a rigorous scientific frame. Thus, recruitment may be affected by selection biases: the survey was limited to responders with internet access and with contacts with local associations. Second, the autistic adult sample may be poorly representative of male autistic adults since most autistic adult responders were females. However, higher responses among females is common for online surveys (Smith, 2008). Third, the autistic adult responders and those represented by carers were not comparable in some of the demographic characteristics. The majority of autistic adults were in or had completed their education at the university/college level. In contrast, 36% of the autistic adults cared for by carers needed a high level of support in daily living and 15% needed institution like-care, indicating a higher need for support in the carers’ adult than the autistic adult responders. Fourth, since the ASDEU survey covered many services areas apart from intervention services, specific clinical information regarding the responders and the intervention was not collected (e.g., the level of social adaptation for each adult, the type of professional who diagnosed the adult and the instrument of diagnosis). Also, we did not collect data on the cultural background of the responders. Indeed, culture may play a role in determining what interventions are available and responses to intervention. While we did not ask for the specific age of starting the intervention, only respondents who had an adult intervention experience in the 2 years prior to taking the survey were eligible to answer the intervention questions. Future studies focusing on intervention services should seek to ask participants for more detailed information about the autism diagnosis, level of social adaptation, cultural, and intervention background. Fifth, medical professionals were less represented in the sample since the majority of professional responders were psychologists or teacher/pedagogues (although this may accurately reflect the profile of adult services professionals in communities). Finally, the highest proportion of responders were those living in Denmark, thus other European communities may be under-represented.

## Summary and Conclusion

The ASDEU survey provides insight into experiences and perceptions of autistic adults, carers, and professionals of services for interventions for autistic adults in 11 European countries. The results illustrate the variation in the degree of alignment between recommended factors for interventions and what is directly experienced by autistic adults, carers, and professionals. Thus, results highlight factors that are closer in alignment to service recommendations, as well as those more likely to be neglected while planning and implementing interventions for autistic adults.

The alignment between real-world experiences and published guidelines was fairly high for the recommended considerations when deciding on an intervention and recommended factors as part of the intervention plan and implementation. Also, in line with the NICE recommendations, psychosocial interventions were experienced by the majority of the responders for core autistic features, daily life skills, co-occurring mental conditions or reducing stress. Pharmacological interventions were experienced mainly for helping to control sleep problems, moods/emotions, or treating co-occurring mental conditions.

The relatively low rate of use of family interventions was somewhat surprising and may reflect an unrecognized need by autistic adults and carers. For professionals, consideration of gender, preferences and consent of the autistic adults when planning an intervention may also warrant further attention.

Few responders that experienced challenging behavior reported receiving an intervention targeted to treat challenging behavior which may also reflect an unmet need. On the positive side, among the three groups, the majority of recommendations regarding factors to consider when planning an intervention for challenging behavior were reported. Pharmacological interventions only and respite care were more often reported to be used by carers of high-level support or institution-like care autistic adults. This may be due by the severity of the adult’s symptoms and the presence of psychiatric or medical co-occurring conditions.

Overall, these results underscore the need to consider the autistic adults’, carers’ and providers’ experience and perceptions when assessing interventions for autistic adults in order to gain a complete view of services needs of a community (Shattuck et al., 2020). In Europe, as in many communities, further development of adult intervention services is a current priority for many stakeholders ranging from autistic adults themselves, their parents and carers, professionals, professional advocacy organizations, and European countries governments and the health and social care sector.

## Supplementary Information

Below is the link to the electronic supplementary material.

Supplementary file1 (DOCX 508 kb)
