# Supplementary material for: Intervention Services for Autistic Adults: An ASDEU Study of Autistic Adults, Carers, and Professionals’ Experiences
Source: J Autism Dev Disord. 2021 May 8;52(4):1623–39. doi: 10.1007/s10803-021-05038-0 (PMC8938388; doi:10.1007/s10803-021-05038-0)
Supplement: Supplementary file 1 — Supplementary file1 (DOCX 508 kb) [file 10803_2021_5038_MOESM1_ESM.docx]

**Supplementary material 1**

*Survey’s questions and answers for autistic adults, carers and professionals*

| Question autistic adult | Question carer | Question professional | Answer autistic adult | Answer carer | Answer professional |
| --- | --- | --- | --- | --- | --- |
| Demographic characteristics | | | | | |
| N/A | N/A | Thinking of your work history, what job or career title fits you best? | N/A | N/A | General practitioner |
|  |  |  |  |  | Psychiatrist |
|  |  |  |  |  | Medical specialist, other than psychiatrist |
|  |  |  |  |  | Nurse |
|  |  |  |  |  | Other medical professional |
|  |  |  |  |  | Psychologist |
|  |  |  |  |  | Social worker |
|  |  |  |  |  | Teacher/pedagogue |
|  |  |  |  |  | Teaching assistant/nursery assistant |
|  |  |  |  |  | Mental health therapist |
|  |  |  |  |  | Physical or occupational therapist |
|  |  |  |  |  | I work in the area of criminal justice (e.g., police, courts, legal advocate) |
|  |  |  |  |  | Other, please specify |
| N/A | N/A | Thinking of your work history, how many years in total have you been in jobs in adult services and care (social, medical, or other services) | N/A | N/A | < 1 year |
|  |  |  |  |  | 1-2 years |
|  |  |  |  |  | 3-5 years |
|  |  |  |  |  | 6-10 years |
|  |  |  |  |  | > 10 years |
| What is your gender? | What is your gender? | What is your gender? | Male | Male | Male |
|  |  |  | Female | Female | Female |
|  |  |  | Other/no answer | Other/no answer | Other/no answer |
| How old are you? | How old are you? | N/A | 18-25 | 18-25 | 18-25 |
|  |  |  | 26-35 | 26-35 | 26-35 |
|  |  |  | 36-45 | 36-45 | 36-45 |
|  |  |  | 46-55 | 46-55 | 46-55 |
|  |  |  | 56-64 | 56-64 | 56-64 |
|  |  |  | > 65 | > 65 | > 65 |
| N/A | How many years of education did you complete? | N/A | N/A | < 10 years | N/A |
|  |  |  |  | 10-12 years |  |
|  |  |  |  | 13-16 years |  |
|  |  |  |  | > 16 years |  |
| Are you going to a school, a home school or in an education program now? | N/A | N/A | Yes, full time | N/A | N/A |
|  |  |  | Yes, part time |  |  |
|  |  |  | No |  |  |
| If no, do you remember your age when you finished your education? | N/A | N/A | Yes | N/A | N/A |
|  |  |  | No |  |  |
| How old were you when you finished your education? | N/A | N/A | _______ | N/A | N/A |
| If yes, what kind of education are you in now? | N/A | N/A | Primary level school | N/A | N/A |
|  |  |  | Secondary level school (for example high school or gymnasium) |  |  |
|  |  |  | Technical, vocational or job training school |  |  |
|  |  |  | College or university |  |  |
| What kind of education were you in when you finished your education: | N/A | N/A | Secondary - level school (for example high school or gymnasium) | N/A | N/A |
|  |  |  | Technical, vocational or job training school |  |  |
|  |  |  | College or university |  |  |
|  |  |  | Don't know |  |  |
|  |  |  | Secondary - level school (for example high school or gymnasium) |  |  |
| Are you: | What is your current employment status now? Pick the answer that fits you best. | N/A | N/A | Student | N/A |
|  |  |  |  | Unemployed |  |
|  |  |  |  | Employed (part time or full time) |  |
|  |  |  |  | Self-employed |  |
|  |  |  |  | Retired |  |
|  |  |  |  | Volunteer |  |
| If you are unemployed, is it because: | N/A | N/A | You are a student | N/A | N/A |
|  |  |  | You are retired |  |  |
|  |  |  | You are looking for a job |  |  |
|  |  |  | You believe that you cannot find a job |  |  |
|  |  |  | You have a disability that prevents you from having a job |  |  |
|  |  |  | Other, please specify |  |  |
| What country do you live in? | What country do you live in? | What country do you work in? | Austria | Austria | Austria |
|  |  |  | Belgium | Belgium | Belgium |
|  |  |  | Bulgaria | Bulgaria | Bulgaria |
|  |  |  | Denmark | Denmark | Denmark |
|  |  |  | England | England | England |
|  |  |  | Finland | Finland | Finland |
|  |  |  | France | France | France |
|  |  |  | Iceland | Iceland | Iceland |
|  |  |  | Italy | Italy | Italy |
|  |  |  | Northern Ireland | Northern Ireland | Northern Ireland |
|  |  |  | Poland | Poland | Poland |
|  |  |  | Portugal | Portugal | Portugal |
|  |  |  | Republic of Ireland | Republic of Ireland | Republic of Ireland |
|  |  |  | Romania | Romania | Romania |
|  |  |  | Scotland | Scotland | Scotland |
|  |  |  | Spain | Spain | Spain |
|  |  |  | Wales | Wales | Wales |
|  |  |  | Other, specify | Other, specify | Other, specify |
| N/A | What is the autistic adult’s gender? | N/A | N/A | Male | N/A |
|  |  |  |  | Female |  |
|  |  |  |  | Other/no answer |  |
| N/A | How old is the autistic adult? | N/A | N/A | 18-25 | N/A |
|  |  |  |  | 26-35 |  |
|  |  |  |  | 36-45 |  |
|  |  |  |  | 46-55 |  |
|  |  |  |  | 56-64 |  |
|  |  |  |  | > 65 |  |
| N/A | How many years have you known the autistic adult? | N/A | N/A | Less than 1 year | N/A |
|  |  |  |  | 1-5 years |  |
|  |  |  |  | 5-10 years |  |
|  |  |  |  | More than 10 years but not the adult's whole life |  |
|  |  |  |  | The adult's whole life |  |
| N/A | How are you related to the autistic adult? | N/A | N/A | Parent | N/A |
|  |  |  |  | Other family member related by blood (not a parent, but for example a child, cousin, grand parent, uncle etc.) |  |
|  |  |  |  | Spouse or partner |  |
|  |  |  |  | A carer, but not a family member, spouse or partner |  |
| Where do you live now? | Where is the autistic adult living now? | Thinking of your current job location where is it located? | Capital city | Capital city | Capital city |
|  |  |  | Other than a capital city | Other than a capital city | Other than a capital city |
| How many people live in the community where you live now? (Answer the best you can) | How many people live in the community where the autistic adult lives now? Answer as best you can | How many people live in the community where your current job is located? | < 1.000 people | < 1.000 people | < 1.000 people |
|  |  |  | 1.000-20.000 people | 1.000-20.000 people | 1.000-20.000 people |
|  |  |  | 20.000-100.000 people | 20.000-100.000 people | 20.000-100.000 people |
|  |  |  | 100.000-1.000.000 | 100.000-1.000.000 | 100.000-1.000.000 |
|  |  |  | > 1.000.000 | > 1.000.000 | > 1.000.000 |
|  |  |  | Don't Know | Don't Know | Don't Know |
| N/A | Pick the description that best fits the autistic adult. | N/A | N/A | Has a high level of independence | N/A |
|  |  |  |  | Has some independence but needs support |  |
|  |  |  |  | Needs a high level of support in daily living |  |
|  |  |  |  | Needs high level institution-like care |  |
| N/A | N/A | The experience and knowledge about services for adults that you have from your current job is: | N/A | N/A | Most closely connected to your current job location (e.g., capital city or small town) |
|  |  |  |  |  | Most closely connected to your current job location and a wider area (e.g., the region or state where your job is located) |
|  |  |  |  |  | Most closely connected to the whole country |
| Interventions for autistic adults | | | | | |
| This section should be answered ONLY if you are in an intervention now or in the last 2 years, such as individual or group therapy to improve life skills or taking medicine for depression | You should answer this section ONLY if you have experience in the last 2 years with an intervention for the autistic adult. For example, the intervention could be individual or group therapy to improve life skills or taking medicine for depression | Do you have knowledge of and current work experience (in the last 2 years) in interventions, such as individual or group therapy or medication, for adults on the autism spectrum? | Yes, I have experience in the last 2 years with an intervention for the autistic adult  No, I don’t have experience in the last 2 years with an intervention for the autistic adult | Yes, I have experience in the last 2 years with the health of the autistic adult. | Yes |
|  |  |  |  | No, I don’t have experience in the last 2 years with the health of the autistic adult | No |
| To answer the following questions, be thinking of the intervention that you are in now or in the last 2 years. If you have had more than one, please, think of the most recent intervention you have had.  Was the intervention started in the last 2 years? | For the following questions, think of the intervention that the adult receives now or in the last 2 years. If the adult has had more than one intervention, please think of the most recent one, when you answer the questions.  Was the intervention started in the last 2 years? | N/A | Yes | Yes | N/A |
| Based on your experience, were the following topics part of the discussion before starting the intervention? | Based on your experience in the last 2 years, were the following topics part of the discussion before starting a new intervention? | Based on your knowledge and work experience in the "area where you work now", how often are the following factors considered when deciding on interventions for adults with autism spectrum? | Your gender (Yes; No; Don’t know) | Gender of the adult (Yes; No; Don’t know) | Gender of the person (Yes; No; Don’t know) |
|  |  |  | Your age | Age of the adult | Age of the person |
|  |  |  | Other kinds of interventions you had in the past | Other kinds of interventions the adult had in the past | History of previous interventions |
|  |  |  | Your experiences in past interventions | How well did the adult do in past interventions | Response to previous interventions |
|  |  |  | N/A | If adult has a learning disability or intellectual disability | Presence of intellectual impairment |
|  |  |  | If you have chronic disorders (e.g., sleep disorder) | If the adult has chronic disorders (e.g., sleep disorder, anxiety) | Presence of other chronic disorders (e.g., sleep disorder, anxiety, sensory issues) |
|  |  |  | What are the kinds of things that seem to be part of the problem to be helped | If there are things that seem to be part of the problem to be helped | Presence of the kinds of things that seem to be part of the problem to be helped |
|  |  |  | What is needed for the intervention, for example people, time, location, payment | What is needed for the intervention, for example, people, time, location, payment | What is needed to implement the intervention (personnel, time, location, payment, etc.) |
|  |  |  | Will you be able to accept or tolerate the intervention | Will the adult be able to accept or tolerate the intervention | If the person will be able to accept or tolerate the intervention |
|  |  |  | Other interventions or treatments already in place | Other interventions or treatments already in place | Other interventions or treatments already in place |
|  |  |  | How well the intervention might work for you | How well the intervention might work for the adult | N/A |
|  |  |  | If you asked for the intervention yourself | If the adult was the person who asked for an intervention | If the person is requesting an intervention |
|  |  |  | How motivated you were to have the intervention | How motivated the adult was to have the intervention | The level of the person’s motivation |
|  |  |  | Your level of stress and well being | The adult's level of stress and well being | The person's level of stress and well being |
|  |  |  | You were asked to give your consent before the intervention started (for example, by signing a consent form) | Whether the adult was asked to give his/her consent before the intervention started (for example, by signing a consent form) | N/A |
| Based on your experience in the last 2 years, after the intervention started was there a regular review to check: | Based on your experience in the last 2 years, after the intervention started was there a regular review to check: | Based on your knowledge and work experience in the "area where you work now", how often are the following factors parts of the intervention plan for an autistic adult? | How much improvement you believed was made? (Yes; No; Don’t know) | How much improvement was made by the adult? (Yes; No; Don’t know) | N/A |
|  |  |  | If you had difficulties with the intervention? | If the adult had difficulties with the intervention? | N/A |
|  |  |  | N/A | N/A | Written protocol for implementing the intervention (Yes; No; Don’t know) |
|  |  |  | N/A | N/A | Method for monitoring and recording any adverse events that occur during the intervention |
|  |  |  | N/A | N/A | Method for monitoring the persons' adherence to the intervention |
|  |  |  | N/A | N/A | Plan for regular review of the benefits and possible challenges of the intervention |
| Were you in a psychosocial intervention in the last 2 years? A psychosocial intervention can be something like individual or group therapy or a support group, but medicines and medical procedures are not part of it. | Has the adult received a psychosocial intervention in the last 2 years? A psychosocial intervention can be something like individual or group therapy or a support group, but medicines and medical procedures are not part of it. | N/A | Yes | Yes | N/A |
|  |  |  | No | No |  |
|  |  |  | Don’t know | Don’t know |  |
| If yes, was the psychosocial intervention for: | If yes, was the psychosocial intervention for: | In this question we are asking about psychosocial interventions (e.g., individualized interventions, group therapy, support groups). Based on your knowledge and work experience in the "area where you work now", how often are psychosocial interventions with autistic adults used for: | Social difficulties (Yes; No; Don’t know) | Core autism symptoms, such as social difficulties (Yes; No; Don’t know) | Core autism spectrum features (Standard, routine practice; Not standard practice, but often considered; Rarely considered; Never considered; Don't know) |
|  |  |  | Daily life skills | Daily life skills | Daily life skills |
|  |  |  | Physical or leisure activity | Physical or leisure activity | Physical or leisure activity |
|  |  |  | Speech and language; communication skills | Speech and language; communication skills | Speech and language; communication skills |
|  |  |  | Treating mental conditions, such as anxiety | Treating mental conditions, such as anxiety | Treating co-morbid mental conditions |
|  |  |  | Improving personal safety | Improving personal safety | Improving personal safety |
|  |  |  | Reducing stress | Reducing stress | Reducing stress |
| Were you in an intervention in the last 2 years that only used medicines and no other kinds of therapy? | Has the adult received an intervention in the last 2 years that only used medicines and no other kinds of therapy? | N/A | Yes | Yes | N/A |
|  |  |  | No | No |  |
|  |  |  | Don’t know | Don’t know |  |
| If yes, was the medicine used for: | If yes, was the medicine used for: | In this question we are asking about pharmacological interventions. Based on your knowledge and work experience in the "area where you work now", how often are pharmacological interventions for autistic adults considered for: | Treating mental conditions, such as depression (Yes; No; Don’t know) | Treating mental conditions, such as depression (Yes; No; Don’t know) | Co-morbid mental conditions (Standard, routine practice; Not standard practice, but often considered; Rarely considered; Never considered; Don't know) |
|  |  |  | Helping to control sleep problems | Helping to control sleep problems | Sleep problems |
|  |  |  | Helping to control moods or emotions (dysregulation) | Helping to control moods or emotions (dysregulation) | Help to control moods or emotions (dysregulation) |
| Were you in an intervention in the last 2 years for treating core autism spectrum symptoms, such as social difficulties, that only used medicines or medical procedures? | Has the adult received an intervention in the last 2 years for treating core autism spectrum symptoms, such as poor social skills, that only used medication or other medical procedures? | N/A | Yes | Yes | N/A |
|  |  |  | No | No |  |
|  |  |  | Don’t know | Don’t know |  |
| If yes, did the medicines or medical procedures include: | If yes, did the medicines or medical procedures include: | In this question we ask about either pharmacological or medical-type interventions used for core autism spectrum behaviors. Based on your knowledge and work experience in the "area where you work now", how often are the following pharmacological or medical-type interventions used for core autism spectrum behaviors specifically: | Medicines for seizures (Yes; No; Don’t know) | Medicines for seizures (Yes; No; Don’t know) | Seizure medication (Standard, routine practice; Not standard practice, but often considered; Rarely considered; Never considered; Don't know) |
|  |  |  | N/A | Medicines for psychosis | Antipsychotics |
|  |  |  | Medicines for depression | Medicines for depression | Antidepressants |
|  |  |  | N/A | N/A | Stimulants |
|  |  |  | Chelation (a kind of treatment to remove metals and minerals from the body) | Chelation (a kind of treatment to remove metals and minerals from the body) | Chelation (chemical treatment to remove metals and minerals from the body) |
|  |  |  | Special diets, food supplements, herbal remedies | Special diets, food supplements, herbal remedies | Special diets, food supplements, herbal remedies |
|  |  |  | Oxytocin | Oxytocin | Oxytocin |
|  |  |  | Secretin | Secretin | Secretin |
|  |  |  | Testosterone | Testosterone | Testosterone |
|  |  |  | Hyperbaric oxygen therapy | Hyperbaric oxygen therapy | Hyperbaric oxygen therapy |
| In the last 2 years, has anyone close to you been in any of the following interventions for family members, siblings, partners or carers of autistic adults? These kinds of interventions help people to better understand autism spectrum and how to better support the autistic adult and themselves. | In the last 2 years, has anyone close to the adult like a family member, sibling, partner or carer received any of the following interventions? These kinds of interventions help people to better understand autism spectrum and how to better support the autistic adult and themselves. | Based on your knowledge and work experience in the "area where you work now", how often are the following interventions available for families, siblings, partners or carers of autistic adults? | Individual therapy (Yes; No; Don’t know) | Individual therapy (Yes; No; Don’t know) | Individual therapy (Standard, routine practice; Not standard practice, but often considered; Rarely considered; Never considered; Don't know) |
|  |  |  | Support groups | Support groups | Support groups |
|  |  |  | Respite care (care for the autistic adult so that their usual carer can have a break) | Respite care (care for the autistic adult so that their usual carer can have a break) | Respite care (care for the autistic adult so that their usual carer can have a break) |
|  |  |  | N/A | N/A | Care planning for the autistic adult |
|  |  |  | N/A | N/A | Advice about care for a person on the autism spectrum |
|  |  |  | Training about autism and planning for your care | Training about autism and planning for your care | Training about autism spectrum |
|  |  |  | Marital counseling (for example, for your spouse, parents or siblings) | Marital counseling | Marital counseling |
| In the last 2 years have you had any behavior where you were harming yourself (such as hitting yourself) or trying to harm yourself (including trying to commit suicide)? | In the last 2 years has the adult had any challenging behavior, for example self-harm, attempted suicide or aggression towards others? | N/A | Yes | Yes | N/A |
|  |  |  | No | No |  |
|  |  |  | Don’t know | Don’t know |  |
| If yes, were you in an intervention to help change the behavior? | If yes, was the autistic adult in an intervention to help change the behavior? | N/A | Yes | Yes | N/A |
|  |  |  | No | No |  |
|  |  |  | Don’t know | Don’t know |  |
| If yes, were any of the following factors talked about with you when deciding on an approach to help change the behavior? | If yes, were any of the following factors talked about when deciding on an approach to help change the challenging behavior? | Based on your knowledge and work experience in the "area where you work now", if an adult on the autism spectrum displays challenging behavior (e.g., self-harm or aggression) how often are the following features considered when deciding on an approach to change the behavior? | If you have a physical disorder (Yes; No; Don’t know) | The presence of a physical disorder (Yes; No; Don’t know) | The presence of a physical disorder (Standard, routine practice; Not standard practice, but often considered; Rarely considered; Never considered; Don't know) |
|  |  |  | If you have difficulties in a personal relationship (e.g., with carer, partner) | Difficulties in personal relationships (e.g., with carer, partner) | Difficulties in personal relationships (e.g., carers, partners) |
|  |  |  | If your physical environment is hard to tolerate (e.g. sensory overload) | Challenges in the physical environment (e.g. sensory overload) | Challenges in the physical environment (e.g. sensory overload) |
|  |  |  | If you have difficulties communicating with others | Communication problems | Communication problems |
|  |  |  | If you have a mental disorder | The presence of a mental disorder, like anxiety | The presence of a mental disorder |
|  |  |  | If you have had recent changes in routine | Recent changes in routine | Recent changes in routine |
|  |  |  | If you have had recent changes in personal circumstances (e.g. loss of someone close) | Recent changes in personal circumstances (e.g. loss of someone close) | Recent changes in personal circumstances (e.g. loss of someone close) |
|  |  |  | The kinds of things that seem to start it or are part of the behavior | Things that could be reinforcing the challenging behavior | Things that could be reinforcing the challenging behavior |
|  |  |  | N/A | Patterns in the challenging behavior | Patterns in the challenging behavior |
|  |  |  | If you have high levels of anxiety | High levels of anxiety | High levels of anxiety |
|  |  |  | If you are in a situation that could be very stressful | Stressful situations | The presence of a situation that could be very stressful |
| Were you in any of the following types of interventions in the last 2 years to help change the behavior? | Has the adult been in any of the following types of interventions in the last 2 years to help change challenging behavior? | Based on your knowledge and work experience in the "area where you work now", how often are the following types of interventions used for challenging behavior in autistic adults: | A psychosocial intervention (something like a support group, individual or group therapy, but no medicines or medical procedures are part of it) (Yes; No; Don’t know) | A psychosocial intervention (something like a support group, individual or group therapy, but no medicines or medical procedures are part of it) (Yes; No; Don’t know) | Psychosocial interventions, only (Standard, routine practice; Not standard practice, but often considered; Rarely considered; Never considered; Don't know) |
|  |  |  | An intervention that combined taking medication with a psychosocial intervention | An intervention that combined medication and psychosocial intervention | Combined psychosocial and pharmacological interventions |
|  |  |  | An intervention that only used medication | An intervention that only used medication | Pharmacological interventions, only |
| In the last 2 years have you had any behavior where you were harming other people (such as hitting other people)? | N/A | N/A | Yes | N/A | N/A |
|  |  |  | No |  |  |
|  |  |  | Don’t know |  |  |
| If yes, were you in an intervention to help change the behavior? | N/A | N/A | Yes | N/A | N/A |
|  |  |  | No |  |  |
|  |  |  | Don’t know |  |  |
| If yes, were any of the following factors talked about with you when deciding on an approach to help change the behavior? | N/A | N/A | If you have a physical disorder (Yes; No; Don’t know) | N/A | N/A |
|  |  |  | If you have difficulties in a personal relationship (e.g., with carer, partner) |  |  |
|  |  |  | If your physical environment is hard to tolerate (e.g. sensory overload) |  |  |
|  |  |  | If you have difficulties communicating with others |  |  |
|  |  |  | If you have a mental disorder |  |  |
|  |  |  | If you have had recent changes in routine |  |  |
|  |  |  | If you have had recent changes in personal circumstances (e.g. loss of someone close) |  |  |
|  |  |  | The kinds of things that seem to start it or are part of the behavior |  |  |
|  |  |  | If you have high levels of anxiety |  |  |
|  |  |  | If you are in a situation that could be very stressful |  |  |
| Were you in any of the following types of interventions in the last 2 years to help change the behavior? | N/A | N/A | A psychosocial intervention (something like individual or group therapy or a support group, but no medicines or medical procedures are part of it) (Yes; No; Don’t know) | N/A | N/A |
|  |  |  | An intervention that combined taking medication with a psychosocial intervention |  |  |
|  |  |  | An intervention that only used medication |  |  |

*Note*. N/A = Question not available for the correspondent group.

**Supplementary Material 2**

*Demographic backgroun*d information of the respondents: adults, carers and professionals

| Characteristic | Answer | Autistic adult  (N=263) |  | Carer / Cared-for adult  (N=302) |  | Professional  (N=132) |
| --- | --- | --- | --- | --- | --- | --- |
| Gender | Female | 180 (68.4) |  | 256 (84.8) / 86 (28.5) |  | 103 (78.0) |
|  | Male | 75 (28.5) |  | 45 (14.9) / 215 (71.2) |  | 27 (20.5) |
|  | Other or no answer | 8 (3.0) |  | 1 (0.3) / 1 (0.3) |  | 2 (1.5) |
| Age (years) | 18-25 | 68 (25.9) |  | 6 (2.0) / 164 (54.3) |  | N/A |
|  | 26-35 | 90 (34.2) |  | 13 (4.3) / 86 (28.5) |  |  |
|  | 36-45 | 62 (23.6) |  | 32 (10.6) / 37 (12.3) |  |  |
|  | 46-55 | 37 (14.1) |  | 117 (38.7) / 13 (4.3) |  |  |
|  | 56-64 | 6 (2.3) |  | 93 (30.8) / 2 (0.7) |  |  |
|  | > 65 | 0 |  | 41 (13.58) / 0 |  |  |
| Living area | | | | | | |
| Country | Denmark | 114 (43.4) |  | 67 (22.2) |  | 17 (12.9) |
|  | France | 33 (12.6) |  | 50 (16.6) |  | 30 (22.7) |
|  | Finland | 41 (15.6) |  | 34 (11.3) |  | 30 (22.7) |
|  | Spain | 13 (4.9) |  | 69 (22.9) |  | 13 (9.9) |
|  | Poland | 17 (6.5) |  | 33 (10.9) |  | 12 (9.1) |
|  | Italy | 18 (6.8) |  | 21 (6.9) |  | 17 (12.9) |
|  | Iceland | 6 (2.3) |  | 23 (7.6) |  | 3 (2.3) |
|  | United Kingdom | 11 (4.2) |  | 0 |  | 8 (6.1) |
|  | Republic of Ireland | 5 (1.9) |  | 5 (1.7) |  | 0 |
|  | German | 4 (1.5) |  | 0 |  | 0 |
|  | Portugal | 0 |  | 0 |  | 2 (1.5) |
| Living area | Capital city | 59 (22.4) |  | 109 (36.1) |  | 44 (33.3) |
|  | Other than a capital city | 204 (77.6) |  | 193 (63.9) |  | 88 (66.7) |
| Community size | < 1.000 people | 15 (5.7) |  | 25 (8.3) |  | 5 (3.8) |
|  | 1.000-20.000 people | 55 (20.9) |  | 62 (20.5) |  | 14 (10.6) |
|  | 20.000-100.000 people | 73 (27.8) |  | 78 (25.8) |  | 38 (28.8) |
|  | 100.000-1.000.000 | 71 (27.0) |  | 79 (26.2) |  | 52 (39.4) |
|  | > 1.000.000 | 25 (9.5) |  | 45 (14.9) |  | 23 (17.4) |
|  | Don't Know | 24 (9.1) |  | 13 (4.3) |  | 0 |
| Education | | | | | | |
| Adult is attending school, home school or education program now | Yes, full time | 38 (14.5) |  | N/A |  | N/A |
|  | Yes, part time | 31 (11.8) |  |  |  |  |
|  | No | 194 (73.8) |  |  |  |  |
| If yes, actual education level | Primary level school | 1 (1.5) |  | N/A |  | N/A |
|  | Secondary level school | 17 (24.6) |  |  |  |  |
|  | Technical, vocational or job training school | 7 (10.1) |  |  |  |  |
|  | College or University | 44 (63.8) |  |  |  |  |
| If no, completed education level | Primary level school | 21 (10.8) |  | N/A |  | N/A |
|  | Secondary level school | 41 (21.1) |  |  |  |  |
|  | Technical, vocational or job training school | 47 (24.2) |  |  |  |  |
|  | College or University | 70 (36.1) |  |  |  |  |
|  | Don't Know | 15 (7.7) |  |  |  |  |
| Adult remember the age when completing education | Yes | 179 (92.3) |  | N/A |  | N/A |
|  | No | 15 (7.7) |  |  |  |  |
| Adult age at completed education | Mean + Standard deviation [range] | 24,1 ± 8.4 [3-52] |  | N/A |  | N/A |
| Carer years of education | < 10 years | N/A |  | 21 (7.0) |  |  |
|  | 10-12 years |  |  | 17 (5.6) |  |  |
|  | 13-16 years |  |  | 73 (24.2) |  |  |
|  | >16 years |  |  | 166 (55.0) |  |  |
|  | Other |  |  | 25 (8.3) |  |  |
| Employment | | | | | | |
| Current employment status | Unemployed | 149 (56.7) |  | 37 (12.3) |  | N/A |
|  | Employed (part time o full time) | 80 (30.4) |  | 159 (52.7) |  |  |
|  | Self-employed | 10 (3.8) |  | 28 (9.3) |  |  |
|  | Student | N/A |  | 5 (1.7) |  |  |
|  | Volunteer | 24 (9.1) |  | 10 (3.3) |  |  |
|  | Retired | N/A |  | 63 (20.9) |  |  |
| If unemployed, reasons for unemployment | A disability that prevents from having a job | 59 (39.6) |  | N/A |  | N/A |
|  | Student | 29 (19.5) |  |  |  |  |
|  | Looking for a job | 11 (7.4) |  |  |  |  |
|  | Believing that she/he cannot find a job | 9 (6.0) |  |  |  |  |
|  | Retired | 9 (6.0) |  |  |  |  |
|  | Other | 32 (21.5) |  |  |  |  |
| Age at diagnosis | | | | | | |
| Age at ASD diagnosis | 16-25 | 44 (24.4) |  | 30 (60.0) |  | N/A |
|  | 26-35 | 61 (33.9) |  | 8 (16.0) |  |  |
|  | 36-45 | 49 (27.2) |  | 10 (20.0) |  |  |
|  | 46-55 | 21 (11.7) |  | 2 (4.0) |  |  |
|  | >=56 | 5 (2.8) |  | 0 (0) |  |  |
| Carers’ relationship with the adult | | | | | | |
| Years of knowledge of the adult | Less than 1 year | N/A |  | 4 (1.3) |  | N/A |
|  | 1-5 years |  |  | 7 (2.3) |  |  |
|  | 5-10 years |  |  | 6 (2.0) |  |  |
|  | More than 10 years but not the adult's whole life |  |  | 21 (7.0) |  |  |
|  | The adult's whole life |  |  | 264 (87.4) |  |  |
| Relationship with the adult | Parent | N/A |  | 258 (85.4) |  | N/A |
|  | Other family member related by blood |  |  | 18 (6.0) |  |  |
|  | Spouse or partner |  |  | 9 (3.0) |  |  |
|  | A carer, but not a family member, spouse or partner |  |  | 17 (5.6) |  |  |
| Level of independence of the ASD adult | High level of independence | N/A |  | 26 (8.6) |  | N/A |
|  | Some independence but needs support |  |  | 122 (40.4) |  |  |
|  | Needs a high level of support in daily living |  |  | 110 (36.4) |  |  |
|  | Needs high level institution-like care |  |  | 44 (14.6) |  |  |
| Professionals’ backgrounds and characteristics of their workplace | | | | | | |
| Knowledge of and current work experience (in the last 2 years) in diagnostic procedures in adults and post-diagnosis support for autistic adults | Yes | N/A |  | N/A |  | 105 (79.6) |
|  | No |  |  |  |  | 27 (20.5) |
| Professional type | Psychologist | N/A |  | N/A |  | 61 (46.2) |
|  | Teacher/pedagogue |  |  |  |  | 17 (12.9) |
|  | Other |  |  |  |  | 15 (11.4) |
|  | Psychiatrist |  |  |  |  | 14 (10.6) |
|  | General practitioner |  |  |  |  | 5 (3.8) |
|  | Social worker |  |  |  |  | 4 (3.0) |
|  | Medical specialist, other than psychiatrist |  |  |  |  | 4 (3.0) |
|  | Physical or occupational therapist |  |  |  |  | 3 (2.3) |
|  | Nurse |  |  |  |  | 3 (2.3) |
|  | Teaching assistant/nursery assistant |  |  |  |  | 3 (2.3) |
|  | Other medical professional (open answer) |  |  |  |  | 2 (1.5) |
|  | Mental health therapist |  |  |  |  | 1 (0.8) |
|  | Criminal justice (e.g. police, courts, legal advocate) |  |  |  |  | 0 |
| Years in jobs in adult services and care | <1 year | N/A |  | N/A |  | 0 |
|  | 1-2 years |  |  |  |  | 0 |
|  | 3-5 years |  |  |  |  | 0 |
|  | 6-10 years |  |  |  |  | 0 |
|  | >10 years |  |  |  |  | 0 |
| Source of experience and knowledge about services for adults | Current job location (e.g., capital city or small town) | N/A |  | N/A |  | 45 (34.1) |
|  | Current job location and a wider area (e.g., the region or state where your job is located) |  |  |  |  | 65 (49.2) |
|  | Most closely connected to the whole country |  |  |  |  | 22 (16.7) |
| Professional knowledge of or current work experience in health conditions, health behaviors and medical contacts in autistic adults | Yes | N/A |  | N/A |  | 113 (65.7) |
|  | No |  |  |  |  | 59 (34.3) |

*Note*. N/A = Question not available for the correspondent group. Values expressed as number of responders and frequencies (in parenthesis).

**Supplementary Material 3**

***Recommended considerations when deciding on an intervention***

|  | Autistic adult  (N=176/177) | | |  | Carer  (N=186) | | |  | Professional  (N=128) | | | | |
| --- | --- | --- | --- | --- | --- | --- | --- | --- | --- | --- | --- | --- | --- |
|  | Yes | No | Do not know |  | Yes | No | Do not know |  | Standard/routine practice | Not standard but often considered | Rarely considered | Never considered | Do not know |
| Presence of things that are part of the problem | 134 (75.7) | 20 (11.3) | 23 (13.0) |  | 132 (71.0) | 34 (18.3) | 20 (10.7) |  | 88 (68.7) | 26 (20.3) | 7 (5.5) | 0 | 7 (5.5) |
| Adult’s level of stress and well-being | 124 (70.4) | 34 (19.3) | 18 (10.2) |  | 139 (74.7) | 32 (17.2) | 15 (8.1) |  | 90 (70.3) | 22 (17.2) | 8 (6.2) | 3 (2.3) | 5 (3.9) |
| The level of the adult’s motivation | 120 (68.2) | 39 (22.2) | 17 (9.7) |  | 103 (55.4) | 55 (29.6) | 28 (15.0) |  | 81 (63.3) | 24 (18.7) | 16 (12.5) | 3 (2.3) | 4 (3.1) |
| Other kinds of interventions had in the past | 110 (62.2) | 39 (22.0) | 28 (15.8) |  | 127 (68.3) | 48 (25.8) | 11 (5.9) |  | 93 (72.7) | 20 (15.6) | 8 (6.2) | 3 (2.3) | 4 (3.1) |
| What is needed for the intervention | 110 (62.5) | 40 (22.7) | 26 (14.8) |  | 123 (66.1) | 47 (25.3) | 16 (8.6) |  | 83 (64.8) | 23 (18.0) | 18 (14.1) | 1 (0.8) | 3 (2.3) |
| How well the intervention might work | 108 (61.4) | 46 (26.1) | 22 (12.5) |  | 111 (59.7) | 48 (25.8) | 27 (14.5) |  | N/A | | | | |
| If the adult will be able to accept the intervention | 104 (59.1) | 46 (26.1) | 26 (14.8) |  | 137 (73.7) | 30 (16.1) | 19 (10.2) |  | 89 (69.5) | 17 (13.3) | 17 (13.3) | 0 | 5 (3.9) |
| If the adult asked for the intervention | 106 (60.2) | 50 (28.4) | 20 (11.4) |  | 64 (34.4) | 106 (57.0) | 16 (8.6) |  | 78 (60.9) | 21 (16.4) | 14 (10.9) | 5 (3.9) | 10 (7.8) |
| Experience in past interventions | 101 (57.1) | 49 (27.7) | 27 (15.3) |  | 113 (60.7) | 50 (26.9) | 23 (12.4) |  | 84 (65.6) | 25 (19.5) | 9 (7.0) | 4 (3.1) | 6 (4.7) |
| Asked to give consent | 97 (55.1) | 52 (29.5) | 27 (15.3) |  | 68 (36.6) | 96 (51.6) | 22 (11.8) |  | N/A | | | | |
| Presence of other chronic conditions | 94 (53.1) | 64 (36.2) | 19 (10.7) |  | 121 (65.1) | 51 (27.4) | 14 (7.5) |  | 94 (73.4) | 23 (18.0) | 7 (5.5) | 0 | 4 (3.1) |
| Age | 91 (51.4) | 63 (35.6) | 23 (13.0) |  | 128 (68.8) | 41 (22.0) | 17 (9.1) |  | 98 (76.6) | 14 (10.9) | 8 (6.2) | 5 (3.9) | 3 (2.3) |
| History or previous interventions | 81 (46.0) | 61 (34.7) | 34 (19.3) |  | 116 (62.3) | 47 (25.3) | 23 (12.4) |  | 81 (63.3) | 27 (21.1) | 12 (9.4) | 0 | 8 (6.3) |
| Gender | 57 (32.2) | 96 (54.2) | 24 (13.6) |  | 79 (42.5) | 84 (45.2) | 23 (12.4) |  | 66 (51.6) | 26 (20.3) | 15 (11.7) | 16 (12.5) | 5 (3.9) |
| Presence of intellectual impairment | N/A | | |  | 118 (63.4) | 53 (28.5) | 15 (8.1) |  | 59 (52.2) | 23 (20.3) | 100 (78.1) | 20 (15.6) | 5 (3.9) |

*Note*. Values expressed as number of responders and frequencies (in parenthesis). N/A=Question not available for the correspondent group. N for the autistic adults varied between 176 and 177. The questions were the following: autistic adult and carer: ‘*Based on your experience in the last two years, were the following topics part of the discussion before starting the intervention?’*; Professional: ‘*Based on your knowledge and work experience in the "area where you work now", how often are the following factors considered when deciding on interventions for adults with autism spectrum?’.*

**Supplementary material 4**

*Number of recommended considerations when deciding on an intervention for autistic adults reported by responders*

| Number of recommendations | Autistic adult  (N=177) | Carer  (N=186) | Professional  (N=144) |
| --- | --- | --- | --- |
|  | *n (%)* | *n (%)* | *n (%)* |
| 0 | 4 (2.3) | 4 (2.2) | 1 (3.5) |
| 1 | 6 (3.4) | 5 (2.7) | 0 (0.0) |
| 2 | 9 (5.1) | 4 (2.2) | 2 (1.6) |
| 3 | 9 (5.1) | 8 (4.3) | 3 (2.3) |
| 4 | 6 (3.4) | 4 (2.2) | 2 (1.6) |
| 5 | 10 (5.7) | 14 (7.5) | 2 (1.6) |
| 6 | 10 (5.7) | 3 (1.6) | 2 (1.6) |
| 7 | 18 (10.2) | 11 (5.9) | 4 (3.1) |
| 8 | 17 (9.6) | 21 (11.3) | 4 (3.1) |
| 9 | 18 (10.2) | 18 (9.7) | 8 (6.3) |
| 10 | 15 (8.5) | 21 (11.3) | 13 (10.2) |
| 11 | 19 (10.7) | 12 (6.5) | 8 (6.3) |
| 12 | 13 (7.3) | 23 (12.4) | 7 (5.5) |
| 13 | 9 (5.1) | 26 (14.0) | 72 (56.3) |
| 14 | 14 (7.9) | 5 (2.7) |  |
| 15 |  | 7 (3.8) |  |

**Supplementary material 5**

***Recommended factors as part of the intervention plan and implementation***

|  | Autistic adult  (N=257) | | |  | Carer  (N=297) | | |  | Professional  (N=127) | | | | |
| --- | --- | --- | --- | --- | --- | --- | --- | --- | --- | --- | --- | --- | --- |
|  | Yes | No | Do not know |  | Yes | No | Do not know |  | Standard/routine practice | Not standard but often considered | Rarely considered | Never considered | Do not know |
| Regular review for improvements/challenges | N/A | | |  | N/A | | |  | 61 (48.0) | 27 (21.3) | 18 (14.2) | 3 (2.4) | 18 (14.2) |
| Written protocol for implementing intervention | N/A | | |  | N/A | | |  | 60 (47.2) | 36 (28.4) | 14 (11.0) | 1 (0.8) | 16 (12.6) |
| Monitoring adherence | N/A | | |  | N/A | | |  | 53 (41.7) | 36 (28.4) | 17 (13.4) | 6 (4.7) | 15 (11.8) |
| Monitoring and recording of adverse events | N/A | | |  | N/A | | |  | 50 (39.4) | 37 (29.1) | 18 (14.2) | 3 (2.4) | 19 (15.0) |
| Improvement made by the adult | 140 (54.5) | 72 (28.0) | 45 (17.5) |  | 186 (62.6) | 88 (29.6) | 23 (7.7) |  | N/A | | | | |
| Adult’s difficulties with intervention | 105 (40.8) | 104 (40.5) | 48 (18.7) |  | 160 (53.9) | 105 (35.4) | 32 (10.8) |  | N/A | | | | |

*Note*. Values expressed as number of responders and frequencies (in parenthesis). N/A =Question not available for the correspondent group. The questions were the following: autistic adult and carer: ‘*Based on your experience in the last 2 years, after the intervention started was there a regular review to check:…’*; professional: ‘*Based on your knowledge and work experience in the "area where you work now", how often are the following factors parts of the intervention plan for an autistic adult?’.*

**Supplementary material 6**

*Number of NOT recommended pharmacological intervention* *for core autistic symptoms in adulthood reported to be experienced by responders*

| Number of recommendations | Autistic adult  (N=255) | Carer  (N=294) | Professional  (N=123) |
| --- | --- | --- | --- |
|  | *n (%)* | *n (%)* | *n (%)* |
| 0 | 219 (85.9) | 235 (79.9) | 24 (19.5) |
| 1 | 6 (2.4) | 4 (1.4) | 15 (12.2) |
| 2 | 20 (7.8) | 24 (8.2) | 18 (14.6) |
| 3 | 8 (3.1) | 25 (8.5) | 28 (22.8) |
| 4 | 2 (0.8) | 5 (1.7) | 23 (18.7) |
| 5 |  | 1 (0.3) | 12 (9.8) |
| 6 |  |  | 1 (0.8) |
| 7 |  |  | 1 (0.8) |
| 8 |  |  | 0 (0.0) |
| 9 |  |  | 1 (0.8) |

**Supplementary material 7**

*Number of recommended criteria to be considered when deciding on an intervention for challenging behavior for autistic adults reported by responders*

| Number of recommendations | Autistic adult – Self harm  (N=38) | Autistic adult – Harm to other  (N=15) | Carer  (N=113) | Professional  (N=119) |
| --- | --- | --- | --- | --- |
|  | *n (%)* |  | *n (%)* | *n (%)* |
| 0 | 0 (0.0) | 1 (6.7) | 9 (8.0) | 7 (5.9) |
| 1 | 1 (2.6) | 1 (6.7) | 1 (0.9) | 0 (0.0) |
| 2 | 0 (0.0) | 0 (0.0) | 5 (4.4) | 0 (0.0) |
| 3 | 2 (5.3) | 1 (6.7) | 7 (6.2) | 0 (0.0) |
| 4 | 1 (2.6) | 1 (6.7) | 5 (4.4) | 0 (0.0) |
| 5 | 3 (7.9) | 1 (6.7) | 13 (11.5) | 4 (3.4) |
| 6 | 4 (10.5) | 3 (20.0) | 9 (8.0) | 2 (1.7) |
| 7 | 7 (18.4) | 1 (6.7) | 9 (8.0) | 1 (0.8) |
| 8 | 7 (18.4) | 3 (20.0) | 15 (13.3) | 4 (3.4) |
| 9 | 8 (21.1) | 3 (20.0) | 6 (5.3) | 3 (2.5) |
| 10 | 5 (13.2) |  | 12 (10.3) | 14 (11.8) |
| 11 |  |  | 22 (19.5) | 84 (70.6) |

**Supplementary Material 8**

*Stratified results of intervention types by high/some independence and high level of support/institution-like care of the a*utistic adult cared reported by carers

| Question | Answer | High or some independence | High-level of support or institution-like care | Total |
| --- | --- | --- | --- | --- |
|  |  | *n (%)* | *n (%)* | *N* |
| Adult received a psychosocial intervention in the last 2 years | Yes | 94 (49.74) | 95 (50.26) | 189 |
| Adult received only pharmacological intervention in the last 2 years | Yes | 47 (37.90) | 77 (62.10) | 124 |
| Adult received only pharmacological intervention for treating core autism spectrum symptoms in the last 2 years | Yes | 22 (36.67) | 38 (63.33) | 60 |
| Family intervention | Individual therapy | 34 (53.97) | 29 (46.03) | 63 |
|  | Training on autism spectrum and care planning | 41 (41.84) | 57 (58.16) | 98 |
|  | Support groups | 38 (50.67) | 37 (49.33) | 75 |
|  | Marital counseling | 11 (73.33) | 4 (26.27) | 15 |
|  | Respite care | 22 (25.29) | 65 (74.71) | 87 |
| Intervention type to change the challenging behavior | Psychosocial interventions, only | 19 (44.19) | 24 (55.81) | 43 |
|  | Psychosocial + pharmacological interventions | 22 (39.29) | 34 (60.71) | 56 |
|  | Pharmacological interventions, only | 12 (32.43) | 25 (67.57) | 37 |
